# A Genomic, Transcriptomic and Proteomic Look at the GE2270 Producer *Planobispora rosea*, an Uncommon Actinomycete

**DOI:** 10.1371/journal.pone.0133705

**Published:** 2015-07-24

**Authors:** Arianna Tocchetti, Roberta Bordoni, Giuseppe Gallo, Luca Petiti, Giorgio Corti, Silke Alt, Joao C. S. Cruz, Anna Maria Salzano, Andrea Scaloni, Anna Maria Puglia, Gianluca De Bellis, Clelia Peano, Stefano Donadio, Margherita Sosio

**Affiliations:** 1 NAICONS Srl, Milano, Italy; 2 KtedoGen Srl, Milano, Italy; 3 Institute of Biomedical Technologies, National Research Council, Segrate, Italy; 4 Dep. STEBICEF, University of Palermo, Palermo, Italy; 5 ISPAAM, National Research Council, Napoli, Italy; Henan Agricultural Univerisity, CHINA

## Abstract

We report the genome sequence of *Planobispora rosea* ATCC 53733, a mycelium-forming soil-dweller belonging to one of the lesser studied genera of *Actinobacteria* and producing the thiopeptide GE2270. The *P*. *rosea* genome presents considerable convergence in gene organization and function with other members in the family *Streptosporangiaceae*, with a significant number (44%) of shared orthologs. Patterns of gene expression in *P*. *rosea* cultures during exponential and stationary phase have been analyzed using whole transcriptome shotgun sequencing and by proteome analysis. Among the differentially abundant proteins, those involved in protein metabolism are particularly represented, including the GE2270-insensitive EF-Tu. Two proteins from the *pbt* cluster, directing GE2270 biosynthesis, slightly increase their abundance values over time. While GE2270 production starts during the exponential phase, most *pbt* genes, as analyzed by qRT-PCR, are down-regulated. The exception is represented by *pbtA*, encoding the precursor peptide of the ribosomally synthesized GE2270, whose expression reached the highest level at the entry into stationary phase.

## Introduction

The genus *Planobispora* is among the lesser studied genera of actinomycetes [1]. Despite the first description in 1968 of the soil isolate *Planobispora longispora* [2], later classified in the family *Streptosporangiaceae* [3], only three additional species have since been validly described: *Planobispora rosea* [4], *Planobispora siamensis* [5] and *Planobispora takensis* [6]. This contrasts with other genera within the *Streptosporangiaceae*: for example, as of 2006, *Streptosporangium* and *Microbispora* had 13 validly described species each [1]. The low number of *Planobispora* species suggests that members of this genus are either not abundant in the natural environments sampled so far and/or difficult to cultivate under laboratory conditions, or that the genus *Planobispora* has limited diversification at the species level.

Nonetheless, *P*. *rosea* ATCC 53733 is relatively well known as the producer of GE2270, the first described thiopeptide targeting elongation factor Tu (EF-Tu) and exhibiting potent activity against Gram-positive pathogens, including methicillin-resistant *Staphylococcus aureus* and vancomycin-resistant *Enterococcus* sp. [7]. Moreover, GE2270 is the only thiopeptide that has yielded semi-synthetic derivatives currently under clinical development, with one compound developed for the treatment of acne [8, 9] and another for *Clostridium difficile* infections [10]. Since *P*. *rosea* ATCC 53733 has been used at industrial scale for the production of GE2270, it is important to gain a better understanding of its physiology in the context of GE2270 biosynthesis.

In this study, we present a genomic, transcriptomic and proteomic analysis of this unusual actinomycete, with particular emphasis on the expression of GE2270 biosynthesis genes. In addition, our analyses show a considerable conservation of the *P*. *rosea* genome in comparison to those of other members of the *Streptosporangiaceae* family.

## Results

### The *P*. *rosea* genome

The genome sequence of *P*. *rosea* ATCC 53733 was carried out using a whole-genome shotgun approach, which resulted in 117 contigs embedded in 10 scaffolds for a total size of 8.69 Mbp and an overall GC content of 70%. The genome size is within the range of other actinomycete genomes and consistent with the physical map derived from pulsed-field gel electrophoresis [11]. Functional annotation identified 8,139 predicted genes corresponding to 8,071 protein-coding genes (designated with the prefix Pros), 4 rRNAs operons and 64 tRNAs. The general properties of the genome are summarized in Table 1.

**Table 1:** Genome features of *P*. *rosea*.

FEATURES	*P*. *rosea*
Length (bp)	8690708
G+C content	~70%
Coding density	88%
Coding sequences (total)	8139
RNAs	68
CDSs	8071
Average CDS length	941

**Fig 1:**
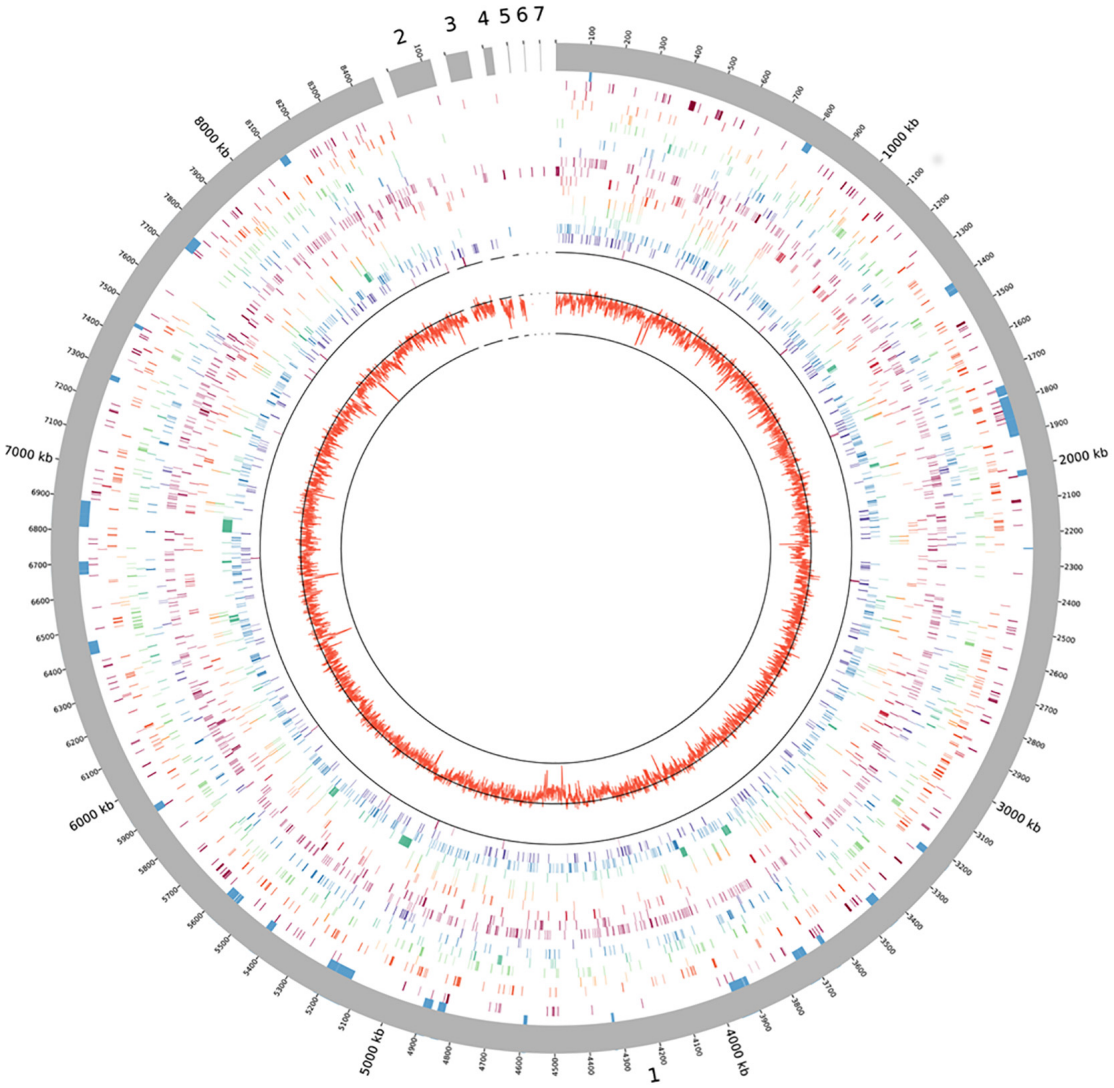
Representation of the *P*. *rosea* genome. Outer grey circle corresponds to the scaffolds and nucleotide length, with the replication origin (*oriC*) placed as nucleotide 1. Blue segments designate secondary metabolite clusters as reported in Table 2. The other circles denote the distribution of CDSs according to the functional categories of S1 Table (from edge to center): C to R, T and U. GC skew is represented by the inner circle.

The four largest *P*. *rosea* scaffolds, which account for 97.4% of the genome, show considerable synteny (see below) with the single-scaffold genome of *Streptosporangium roseum* NRRL B-2638 [12]. The *P*. *rosea* scaffolds were thus *in silico* ordered using the latter genome as template, creating an 8,466,550-bp superscaffold. The remaining 6 scaffolds, which overall account for 224,158 bp, could not be ordered on the basis of synteny with *S*. *roseum* and were arbitrarily placed at the end of the superscaffold, resulting in the draft genome depicted in Fig 1.

**Fig 2:**
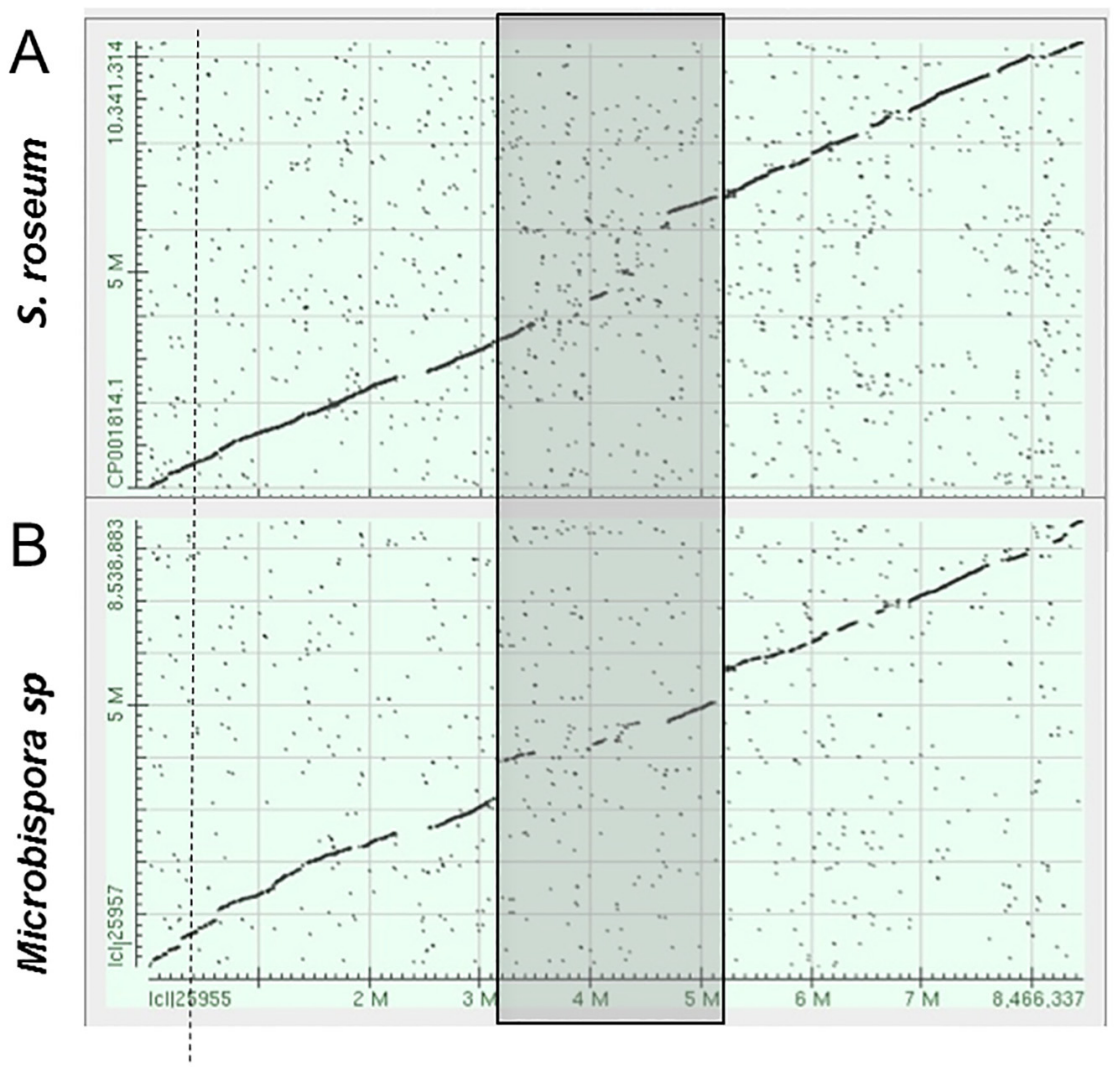
Synteny of the *P*. *rosea* genome in comparison with *S*. *roseum* (panel A) and *Microbispora* sp. (panel B). Dots represent reciprocal best hits obtained by pairwise BlastN searches. Numbers indicate genome coordinates in Mbp, with *dnaA* located at nucleotide 1 for each genome. The shaded box refers to the divergent segment in the *P*. *rosea* genome, where clusters 7 through 13 (Table 2) are located. The dashed vertical line denotes the position of the *pbt* cluster.

The 9,389- and the 7,977-gene genomes of *S*. *roseum* [12] and *Microbispora* sp. ATCC PTA-5024 [13], respectively, were compared to the 8,139-gene *P*. *rosea* genome. Apart from a central 1-Mbp region, the *P*.*rosea* genome appears to be closely related to that of *S*. *roseum* (Fig 2A) and of *Microbispora* (Fig 2B). In contrast, just a few syntenic regions are observed with the *Streptomyces coelicolor* genome (S1 Fig). While the genome divergence with *S*. *coelicolor* is expected for strains belonging to the different orders *Streptosporangiales* and *Streptomycetales* within the *Actinobacteria* [14], the high relatedness between the *Planobispora*, *Streptosporangium* and *Microbispora* genomes warranted further investigations.

**Fig 3:**
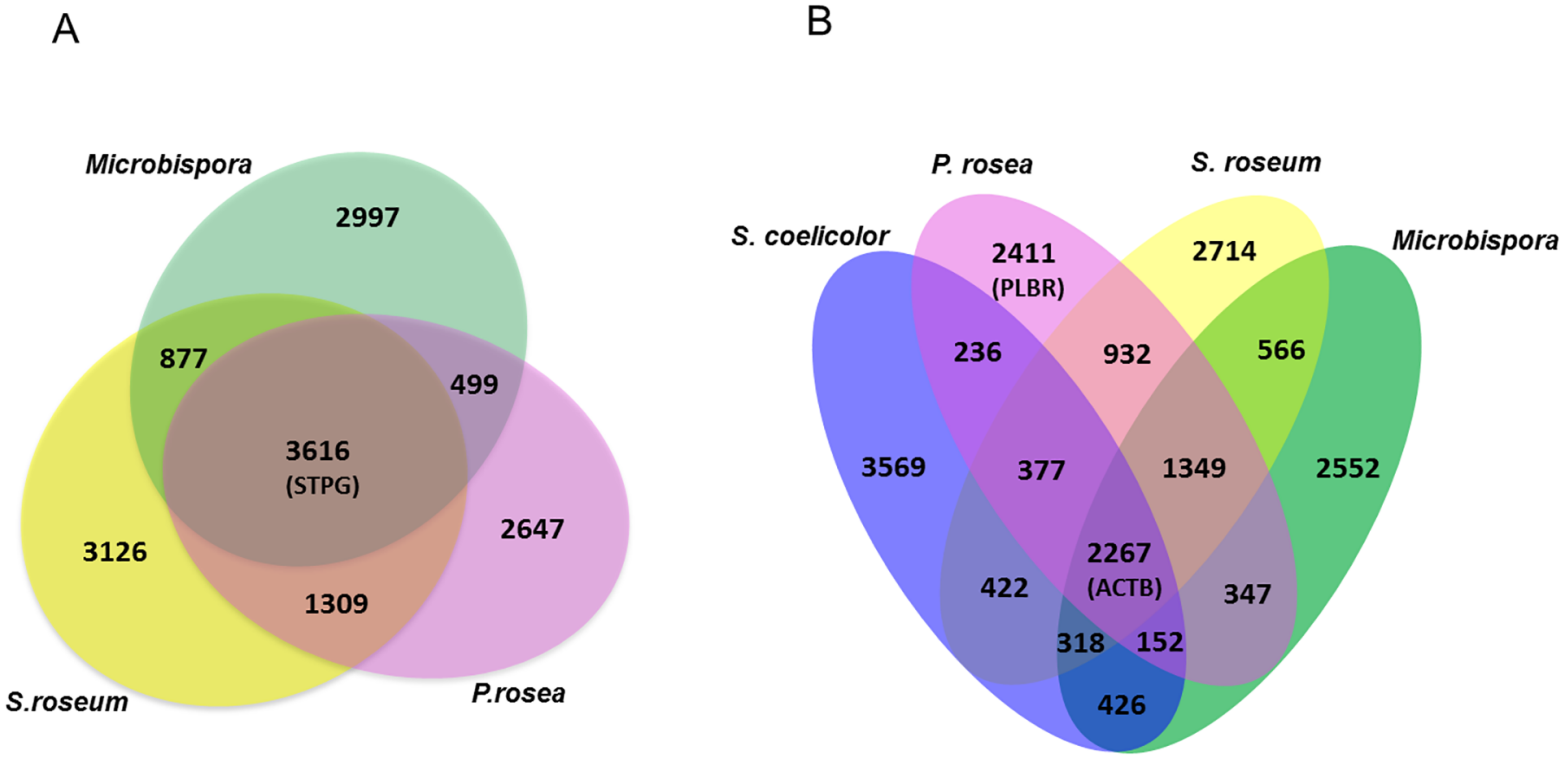
Shared CDSs among selected genomes. The Venn diagrams represent the number of orthologs found in the *P*. *rosea*, *S*. *roseum* and *Microbispora* genomes (A) or in these three genomes and *S*. *coelicolor* (B). "STPG" refers to the orthologs shared by the three *Streptosporangiaceae* genomes; "ACTB" to the orthologs common also with *S*. *coelicolor*; and "PLBR" to the unique *P*. *rosea* genes.

Using the OrthoMCL algorithm, the protein-coding genes of the three *Streptosporangiaceae* strains were grouped in the Venn diagram of Fig 3A: a considerable number (3,616) of orthologs are shared, which account for 38–44% of each genome. According to this analysis, *P*. *rosea* appears to be more related to *S*. *roseum*, with which it shares a further 1,309 genes, than to *Microbispora*, with just an additional 499 genes in common. When this analysis was extended to *S*. *coelicolor*, the number of shared orthologs drops to 2,267 (Fig 3B). Thus, members of the *Streptosporangiaceae* differ from the model *Streptomyces* species not only in overall chromosome organization (as shown for *P*. *rosea* in S1 Fig), but also in gene composition (Fig 3B). Only a small number of orthologs are shared between *S*. *coelicolor* and just one of the analyzed genomes: 236, 422 and 426 with *P*. *rosea*, *S*. *roseum* and *Microbispora*, respectively (Fig 3B). Overall, each of the *Streptosporangiaceae* genome harbors between 2,647 and 3,126 unique genes (Fig 3A). We refer to the orthologs shared by the three *Streptosporangiaceae* genomes as “STPG”; of these, the 2,267 orthologs common also with *S*. *coelicolor* are designated as “ACTB”; while the 2,411 unique *P*. *rosea* genes are “PLBR”.

A subdivision into functional categories of the STPG, ACTB and PLBR genes did not highlight any significant differences in relative abundance of specific categories (S1 Table). The only exception is represented by *Unclassified*, with 1515, 768 and 302 CDSs falling in the PLBR, STPG and ACTB groups, respectively. Accordingly, a significant majority (63%) of the PLBR sequence are *Unclassified* while, as predicted, only 13% of the ACTB genes belong to this category. As expected, COGs directly related to primary metabolism are enriched within the STPG and ACTB groups. It is worth mentioning that most of the genes under *Cell motility* fall within the PLBR group.

### Gene clusters involved in secondary metabolism

Actinomycetes have the genomic potential to produce more secondary metabolites than those usually identified under laboratory conditions [15]. Using signature oligonucleotides for nonribosomal peptide synthetase (NRPS) genes, we previously reported the presence of 13 distinct NRPS gene clusters in the *P*. *rosea* genome [16]. Consistent with this early observation, running the *P*. *rosea* genome on the antiSMASH platform [17] led to the identification of 26 distinct gene clusters directing the synthesis of secondary metabolites, including 11 NRPS-encoding clusters (Table 2). In addition to the recently characterized *pbt* cluster, responsible for the ribosomally synthesized GE2270 [18], the list of Table 2 includes clusters for the biosynthesis of as-yet-uncharacterized polyketides, ribosomal and non-ribosomally synthesized peptides, siderophores, bacteriocins and terpenes. All together, these 26 clusters represent ca. 12% of the *P*. *rosea* genome and highlight the considerable biosynthetic potential of this strain for producing bioactive metabolites, as hinted by previous studies [16].

**Table 2:** List of *P*. *rosea* clusters identified by Antismash.

Cluster number	Span (nt)^a^	Length (bp)	Span CDS (Pros_)^a^	compound class^b^	related cluster^c^	
					*in S*. *roseum* ^*d*^	*in Microbispora* ^*e*^
**1**	102,380–109,761	11,862	0100–0109	bacteriocin	-	-
**2**	791,027–812,448	38,036	0802–0818	**GE2270**	-	-
**3**	1,403,938–1,438,390	65,523	1361–1384	NRP	4,173,990–4,201,747	-
**4**	1,858,372–1,906,489	53,045	1763–1796	NRP-PK(T1)-lanthipeptide		-
**5**	2,010,264–2,027,988	45,219	1902–1918	NRP		**2,371,414–2,403,492**
**6**	2,247,974–2,251,793	10,215	2120–2127	bacteriocin	-	-
**7**	3,219,426–3,239,019	20,881	3078–3094	terpene	**3,475,138–3,490,940**	-
**8**	3,431,409–3,461,415	44,541	3272–3293	NRP	-	-
**9**	3,639,023–3,652,991	13,872	3480–3490	bacteriocin	-	-
**10**	3,703,411–3,741,769	57,560	3525–3543	NRP	-	-
**11**	3,898,951–3,956,216	61,986	3694–3722	NRP-PK(T1)	-	-
**12**	4,320,599–4,329,651	20,908	4097–4104	terpene	4,266,350–4,274,478	5,063,534–5,071,621
**13**	4,583,945–4,595,355	22,198	4339–4349	terpene	-	
**14**	4,835,465–4,858,142	32,732	4573–4597	lanthipeptide	**6,500,452–6,518,122**	
**15**	4,875,676–4,904,714	41,073	4613–4637	PK(T3)	**6,518,284–6,541,718**	**4,846,450–4,875,745**
**16**	5,132,116–5,214,926	88,968	4850–4889	NRP-PKS(T1)	-	-
**17**	5,413,815–5,432,644	22,669	5076–5090	lanthipeptide	-	-
**18**	5,541,672–5,587,353	69,615	5194–5217	NRP		-
**19**	5,911,736–5,933,802	20,998	5547–5561	terpene	**7,547,237–7,571,380**	**6,130,177–6,152,363**
**20**	6,421,574–6,464,257	68,013	5997–6024	NRP	-	-
**21**	6,667,875–6,706,140	49,061	6201–6232	NRP	-	-
**22**	6,811,466–6,890,589	94,011	6336–6364	PK(T1)	-	-
**23**	7,264,643–7,282,898	13,373	6710–6721	siderophore	**9,224,692–9,248,620**	**7,334,898–7,349,092**
**24**	7,439,714–7,450,733	10,806	6872–6882	bacteriocin	-	**7,514,673–7,526,493**
**25**	7,719,251–7,762,437	46,458	7132–7162	PK(T1)	**9,703,532–9,749,956**	-
**26**	8,110,653–8,136,057	56,403	7499–7507	NRP		-

Clusters of secondary metabolites identified by Antismash [17] in *P*. *rosea*, putative products and their conservation in *S*. *roseum or Microbispora*. Coordinates refer to the *S*. *roseum* [12] and *Microbispora* sp. genome [13]

^a^ as identified by Antismash followed by manual curation;

^b^ as identified by Antismash except for cluster 2 which corresponds to GE2270 cluster;

^c^ as defined under Methods. Entries in bold type refer to equivalent genome position. Cluster for secondary metabolites were considered invariant when they shared CDSs involved in the structural scaffold and at least 50% of additional CDSs.

The 26 gene clusters are located along the chromosome, with some relative abundance in the central, non-conserved region (Figs 1 and 2). Consistent with being embedded within genes coding for DNA-directed RNA polymerase subunits and for components of the translation machinery [18, 19], the *pbt* cluster (S2 Fig) is located around 0.8 Mbp in a highly conserved region of the genome (Fig 2).

Because of the high relatedness of the *P*. *rosea*, *S*. *roseum* and *Microbispora* sp. genomes (Fig 2), we wondered whether the three strains shared also some secondary metabolite clusters. Five out of the 21 *Microbispora* sp. clusters present identical gene composition and equivalent chromosomal location with corresponding clusters of *P*. *rosea* (Table 2). A similar comparison with the 25 *S*. *roseum* clusters led to the identification of six shared clusters (Table 2). Three of these clusters are present at equivalent positions in all three genomes: they specify synthesis of a terpene, of a polyketide and of a siderophore. It remains to be seen whether any of these shared clusters are involved in essential cellular functions. As expected from the genomic divergence mentioned above, no *P*. *rosea* cluster is shared with *S*. *coelicolor*.

### *P*. *rosea* growth and GE2270 production kinetics

**Fig 4:**
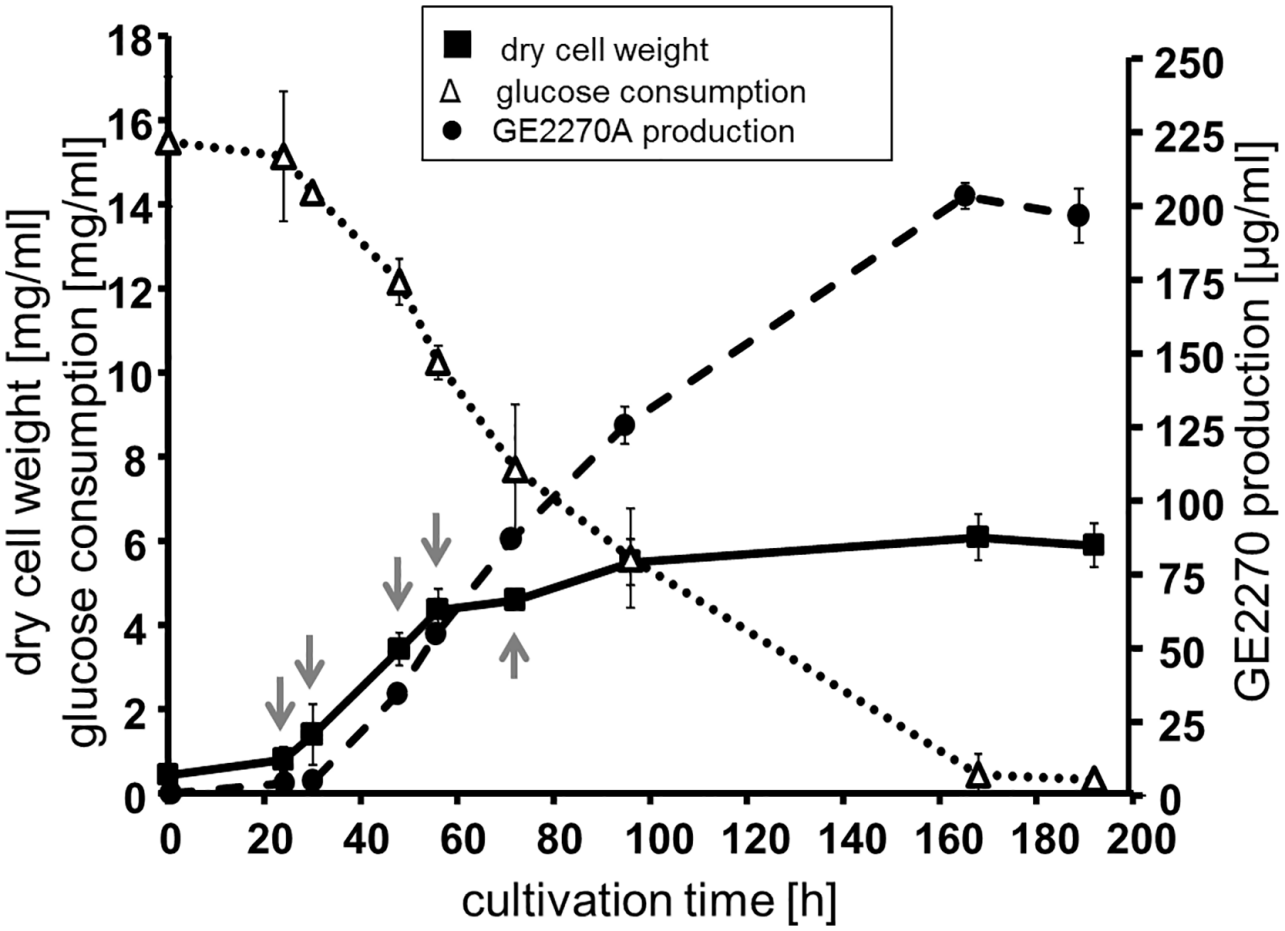
*P*. *rosea* growth, glucose consumption and GE2270 production. Flask cultivation of *P*. *rosea* was performed in V6 medium. Biomass accumulation was calculated from dry cell weight measurements, while GE2270A production was obtained after whole culture extraction. Each point represents the mean and standard deviation of three independent cultures. The arrows indicate the time points of sample collection for transcriptomic and proteomic analyses. See Materials and Methods for experimental details.

When *P*. *rosea* was grown in complex medium, exponential growth started about 24 h after inoculum and proceeded up to 54 h, when biomass accumulation increased slightly (Fig 4). As previously observed [18], production of GE2270A started during the first stages of exponential growth and continued until 168 h. An almost linear production was observed peaking around 200 μg/ml, and further product accumulation appeared to coincide with glucose exhaustion.

Samples for comparative proteome and RNA-Seq analyses were collected at 24 (around onset of the exponential growth phase), 48 (around the end of the exponential growth phase) and 72 h (during stationary growth phase but sustained GE2270 production). For RNA-Seq analysis, additional samples were collected at the early stages of the exponential growth phase (30 h) and at the transition to slower growth (54 h).

### Differential proteome analysis

Four biological replicas were used for each time point and a total of 1,789 reproducible protein spots were obtained in 2D-gel maps. The observed 2D-distribution pattern of protein spots was similar to the theoretical 2D proteome derived from the *P*. *rosea* genome (S3 Fig). Protein spot abundances were comparatively evaluated using as reference the 24-h abundance values and considering as differentially abundant those spots showing a ≥1.5 fold increase or decrease with respect to the reference value in a statistically significant manner (i. e. P≤ 0.05 in ANOVA test). Accordingly, 64 and 109 protein spots were differentially abundant at 48 and 72 h, respectively, with 47 spots in common between the two time points, leading to a total of 126 protein spots. These spots, identified by MS and assigned to specific CDSs from the *P*. *rosea* genome, distributed into 16 COG classes (S2 Table; S4 Fig) according to the function annotation tool at the WebMGA server. Excluding spots corresponding to multiple protein identification (S2 Table), “Energy production and conversion” (C), “Translation, ribosomal structure and biogenesis” (J), and “Unclassified” were the most represented classes (S4 Fig).

The abundance ratios of the identified protein spots were categorized as *C*, *D* or *I* for constant (i.e. no significant change with respect to the 24-h value), decreased or increased, respectively. Thus, the differentially abundant protein species could be categorized into six possible groups: *DD*, *CD*, *ID*, *DC*, *DI*, *CI* and *II*, where the first and second positions refer to 48- and 72-h category, respectively (S2 Table).

In order to highlight possible global changes in cell metabolism and molecular processes, the abundance profiles of protein species were distributed into the corresponding COG classes (S4 Fig): the functional categories associated with primary anabolic and catabolic processes (C, E, G and M) and macromolecular synthesis (J, K and L) mostly decrease their abundance over time (S4 Fig), in accordance with the cells entering the stationary growth phase.

**Fig 5:**
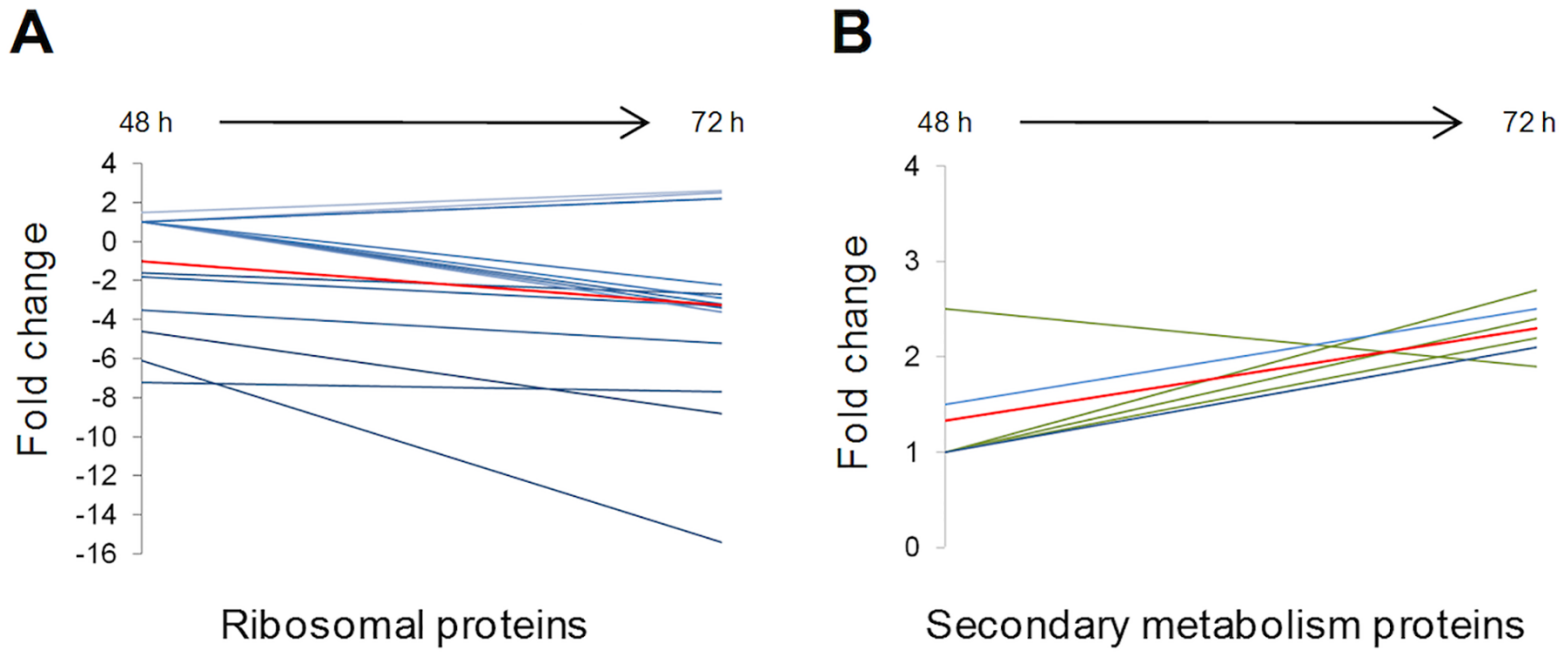
Abundance trends of ribosomal (A) and secondary metabolism (B) proteins. Mean trend of each group is reported as red line and relative abundance of single protein species is showed as light blue line with the exception of abundance trends of *ptb* gene products which are reported as green line in panel B.

In particular, among the ribosomal proteins (J class) showing a decreasing abundance trend overtime (Fig 5), Pros_0822, the GE2270-insensitive EF-Tu [20] has a *CD* profile (S2 Table). On the other hand, protein species from four CDSs associated with secondary metabolism (Table 2) showed an increasing accumulation trend (Fig 5B; S2 Table). Among them, PtbM3 (Pros_0817) and PbtM1 (Pros_0807) from *pbt* gene cluster possessed a *II* and *CI* profile, respectively. Pros_0817 and Pros_0807 encode a radical SAM enzyme for C-methylation of thiazole D and an N-methyltransferase for N-methylation of Asn3 [19], respectively.

Among the observed differentially expressed proteins, 34 belong to the ACTB, 4 to the PLBR and 5 to the STPG group (S2 Table), thus revealing that most of protein regulation associated to the transition from exponential to stationary growth phase affects conserved gene products. It is noteworthy that ACTB CDS products are mostly proteins participating into primary metabolism and macromolecular synthesis while PLBR CDS products are in prevalence secondary metabolism proteins. The identified STPG products are essentially hypothetical or poorly characterized proteins.

### RNA-Seq analysis

RNA samples were extracted at the different time points from two parallel cultures and processed separately for sequencing to have two replicates for each bacterial culture. The resulting sequence reads were mapped on the draft *P*. *rosea* genome and an average sequencing depth of 45X was obtained, with more than 90% of annotated CDSs covered by at least 10 reads in all analyzed time points (S3 Table).

Data were analyzed to identify differentially expressed genes (DEGs) in comparison with their levels at 24 h. Considering each time point as an independent entry and setting the confidence threshold at p-value ≤ 0.01 and FoldChange value ≤ -1 and ≥ 1, 297, 426, 457 and 555 DEGs were identified at 30, 48, 54 and 72 h, respectively (S4 Table). Overall, a total of 1,029 genes, or 13% of the *P*. *rosea* CDSs, alter their expression levels under these experimental conditions. Most of the DEGs at 30, 48 and 54 h were down-regulated (91, 72 and 90%, respectively), while at 72 h we observed an equivalent number of up- and down-regulated DEGs. This result is consistent with the predicted difference in physiological conditions between 72 h (entry into stationary phase) and the onset of exponential phase.

Among the four lists of DEGs, 202 genes were significantly and consistently differentially expressed along the entire time course. These DEGs could be divided into two main clusters, with most of them (196 DEGs) showing down-regulation after 24 h (S5 Fig) and the remaining DEGs showing up-regulation after 48 h (S5 Fig and S5 Table).

Enrichment of particular functional categories was analyzed by applying a Fisher Test to all the single two-class comparisons. Categories C (*Energy production* and *conversion*) and O (*Posttranslational modification*, *protein turnover*) were significantly enriched (adjusted p-value <0.01) in the first three coupled-call comparisons (i.e. 24 vs 30h; 24 vs 48h; and 24 vs 54h), thus the corresponding genes are the most down-regulated during the shift from exponential phase. These two functional categories are the main contributors to the transcriptional profile changes observed during the growth curve. Among the 555 72-h DEGs, 50% (276) of them belong to the STPG group, with most (182 DEGs) showing down-regulation at 72 h. Conversely, most (113 out of 154 DEGs) of the differentially expressed PLBR genes are upregulated at 72 h.

### Expression of genes for secondary metabolism

**Fig 6:**
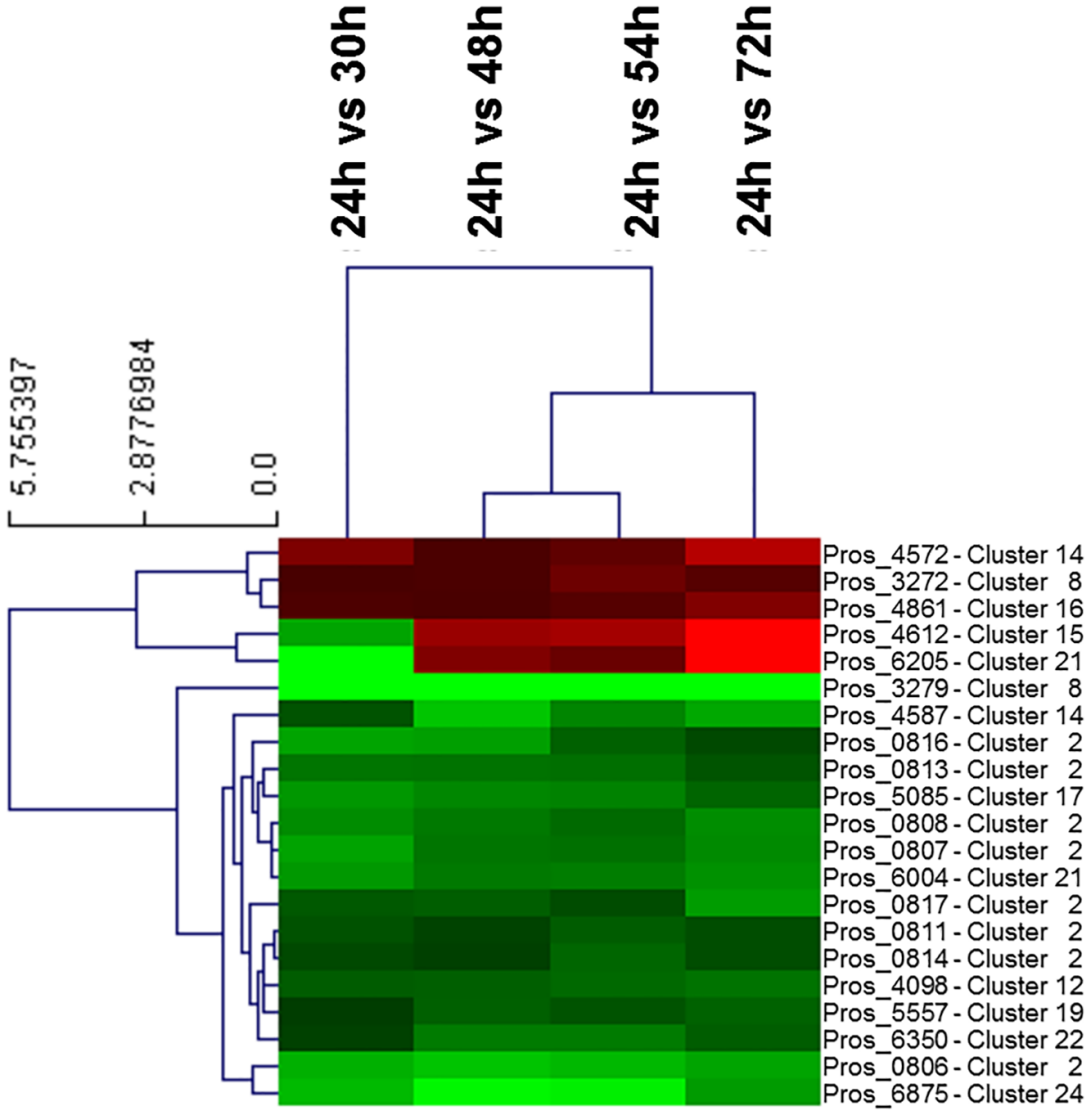
Trends of selected DEGs. HeatMap representation of differentially expressed genes from Table 2 with p-value<0.05. Genes were hierarchically clustered at sample and gene level on the basis of Euclidean distances (left hand side of the figure). The correspondence of each gene to the clusters of Table 2 is also shown.

As mentioned above, the *P*. *rosea* genome harbors 26 predicted clusters (Table 2), for a total of 528 genes, 464 of which were covered by at least 10 RNAseq reads in all the analyzed growth conditions. Their expression profiles were analyzed by applying a hierarchical clustering at sample and gene level based on Euclidean distance (S6 Fig). As a result, genes were categorized as follows: those with a trend of down-regulation after 30 h; a small group with genes up-regulated after 48 h; those up-regulated after 30 h; and those with slight trends of down- or up-regulation after 30 h. Notably, the *pbt* cluster is highly represented within the first group, with 10 out of 17 genes down-regulated after 30 h. Clusters 14 and 24 show a *pbt*-like trend of down regulation. Notably, each of these two clusters, encoding a lanthipeptide and a bacteriocin, respectively, is shared with *S*. *roseum* and *Microbispora* sp. (Table 2). When this analysis was limited to those genes differentially expressed with a p-value <0.05, only the first three groups of expression trends were found, and genes belonging to the same cluster continued to be differentially expressed (Fig 6).

### The transcription profile of the *pbt* cluster

We next focused our attention on the *pbt* genes (Pros_0802 to Pros_0818), as well as the cluster-flanking genes Pros_0799 and Pros_0800, encoding the RNA polymerase β and β’ subunits, respectively, and Pros_0819 to Pros_0822, encoding components of the protein synthesis apparatus (S2 Fig). The RNA-Seq analysis indicated that most of the *pbt* genes are characterized by a strong down-regulation after 30 h maintained until 72 h (Fig 6; S4 Table), with the highest expression occurring at the onset of the log phase. The notable exception to this synchronized behavior was represented by Pros_0809 (*pbtA*), slightly down-regulated after 30 h and up-regulated at 72 h.

**Fig 7:**
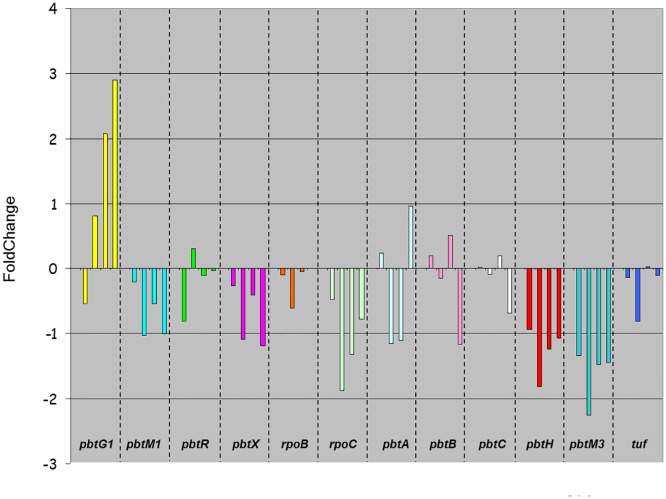
qRT-PCR of selected *pbt* genes. The figure reports the relative FoldChange at 30, 48, 54 and 72 h (in order, left to right) relative to 24 h for each analyzed gene.

qRT-PCR analysis of the most differentially expressed *pbt* genes, along with cluster-flanking genes, indicated an adequate correlation with transcriptome data, as exemplified in S7 Fig. Indeed, the expression levels of *rpoB and C*, *pbtR*, *pbtX*, *pbtM1*, *pbtB*, *pbtC*, *pbtH*, *pbtM3*, and *tuf* (Pros_799 _800, _802, _806, _807, _810, _811, _816, _817, and _822, respectively) were highest at 24 h and declined afterwards (Fig 7), confirming that most *pbt* genes are up-regulated at the onset of the log phase and undergo an overall down-regulation after 30 h. Notable exceptions to this trend are represented by *pbtA*, whose expression was strongly upregulated at 72 h, and by *pbtG1*, encoding a cyclodehydratase, which appears to be upregulated along the growth curve (Fig 7).

## Discussion

The *P*. *rosea* genome presents considerable convergence in gene organization and function with the available genomes from two other members in the family *Streptosporangiaceae*, *Streptosporangium roseum* and *Microbispora* sp. The three strains share a significant number of orthologs, but only 62% of them are shared also with the model actinomycete *Streptomyces coelicolor*. When the four genomes were compared in terms of functional categories, no significant differences in relative abundance were observed between the SPTG and the ACTB orthologs, indicating that the differences are associated with many cellular functions. It is interesting to point out that *P*. *rosea* differs significantly from the other two *Streptosporangiales* in the number of unique genes associated with category N, *Cell motility* (S1 Table). It is tempting to speculate that this feature is related to the fact that the genus *Planobispora* is endowed with motile spores, a feature associated with neither *Streptosporangium* nor *Microbispora* [1].

Another unexpected finding from the genomic analysis is the existence of a significant number of gene clusters for secondary metabolism shared with the other two *Streptosporangiales* genomes and often present in the same chromosomal location. In contrast, most *Streptomyces* strains do not share a significant number of gene clusters [21]. Nonetheless, *P*. *rosea* harbors a considerable genetic potential to produce secondary metabolites, with most biosynthetic classes represented by at least one cluster.

*P*. *rosea* is important as the producer of the thiopeptide GE2270, with derivatives thereof currently under clinical development. Remarkably, the *pbt* cluster lies in a core region of the genome, next to housekeeping genes encoding components of the transcription and translation apparatus. Nonetheless, this cluster is not present in the other two *Streptosporangiales* strains. Consistent with its chromosomal location, the *pbt* genes are expressed during the exponential phase, and their expressione levels generally declines thereafter along with other housekeeping genes. That GE2270 production is hard-wired with growth is consistent with the observation that glucose consumption parallels GE2270 production, even without any apparent increase in biomass. Notably, some of the Pbt proteins were readily detected as differentially expressed proteins.

The exception to this general expression trend is represented by *pbtA*, encoding the precursor peptide of GE2270, whose expression reached the highest level at the entry into stationary phase, and by *pbtG1*, which has been proposed to govern a biosynthetic checkpoint during precursor peptide maturation [18]. Since GE2270 accumulation requires the transformation of the precursor peptide PbtA in the finished thiopeptide, an increase in GE2270 concentration with time is likely to require a continuous supply of the precursor, hence a sustained expression of *pbtA*.

The observation that members of the *Streptosporangiaceae* are highly divergent from the model actinomycete *S*. *coelicolor*, but quite related among each other, may have important implications for the industrial use of strains from this family. Indeed, their industrial importance is increasing. For example, the recently approved glycopeptide dalbavancin is obtained by conversion of the glycopeptide A40926, produced by *Nonomuraea* sp. [22]; while compounds produced by *Microbispora*, *Nonomuraea* or *Planobispora* itself are under late preclinical/early clinical studies [23]. The results reported herein represent a starting point for a better understanding of the physiology of *Planobispora rosea* and of other *Streptosporangiaceae*, which may help in the design of improved production processes.

## Materials and Methods

### Media and growth conditions

A frozen inoculum of *P*. *rosea* ATCC 53733 was routinely pre-cultivated in 10 ml D-Seed (20 g/L starch, 5 g/L peptone, 3 g/L yeast extract, 2 g/L meat extract, 2 g/L soybean meal, 1 g/L calcium carbonate; pH 7.0) at 30°C for 2 days at 200 rpm. Next, a 10% inoculum was made in V6 medium (20 g/L dextrose, 5 g/L yeast extract, 5 g/L meat extract, 5 g/L peptone, 3 g/L hydrolizated casein, 1.5 g/L NaCl; pH 7.3) at 30°C for 9 days at 200 rpm. For the preparation of frozen stocks, the cells of a D-Seed pre-culture were harvested by centrifugation at an OD_600_ of 0.7. The cells were resuspended with fresh D-seed medium (in 1/5 of the original culture volume) and stored at -80°C in 1 ml aliquots.

Dry cell weight at different time points was used to determine biomass accumulation. Accordingly, 3 mL from three parallel flasks were combined, and 7 mL thereof were filtered through pre-weighted membrane filters (0.45 μm pore size). The filters were dried at 60°C to constant weight.

For the determination of GE2270 production, 1 ml culture was extracted with an equal volume of ethyl acetate. The organic phase was removed and, after evaporation of the solvent, the residue was dissolved in 100 μl methanol. Ten μl were analysed by HPLC (LC 2010A-HT; Shimadzu) with a LiChrosphere C18-5 column (100 x 4.6, 5 μm; Merck) at a flow rate of 1 mL/ min using a linear gradient from 30 to 100% phase B over 30 min. Phase A was 0.1% TFA (v/v) in water and phase B was acetonitrile. UV detection was carried out at 225 nm. Authentic GE2270A was used as standard. Under these experimental conditions, >80% of the GE2270 complex consisted of congener A.

Glucose consumption was measured with a GM8 Micro-State (Analox Instruments) according to the manufacturer instructions, using 20 g/ L glucose as standard.

### DNA extraction, sequencing and genome assembly

Genomic DNA was extracted from *P*. *rosea* as follows: 5 ml culture was washed with 10 ml 0.3 M sucrose, resuspended in 5 ml of 75 mM NaCl, 25 mM EDTA, 20 mM TrisHCl pH 7.5 containing 20 mg/ml lysozyme (Sigma-Aldrich) and incubated at 37°C. Fresh lysozyme (20 mg/ml) was added every 8–12h, 25 μg ribonuclease A (Sigma-Aldrich) was added after 24 h, and 3 mg proteinase K (Sigma-Aldrich) after 28 h. After a 30-h incubation, SDS was added to 1% and the mixture incubated overnight at 55°C. After addition of 2 ml 5M NaCl, the mixture was extracted with 4 ml phenol-chloroform-isoamylalcohol and then with 4 ml chloroform. After precipitation with ice-cold isopropanol, DNA was spooled using a sealed glass Pasteur pipette and washed in 70% ethanol. The DNA was air-dried and resuspended in 10 mM Tris-HCl, 1 mM EDTA pH 8.0.

Genomic sequence was performed using a high-throughput automated DNA sequencing platform (454 GS FLX Instrument, Roche) following the manufacturer's procedures [24]. The library for genomic DNA sequencing was prepared according to the Rapid Library Preparation protocol (454, Roche). Briefly, after shearing genomic DNA by nebulization, the ligation to specific adaptors and the preparation of the fragmented DNA library were performed. The DNA library was then amplified in an emulsion-based clonal reaction. After recovery and washing, the library fragments were loaded onto a PicoTiterPlate and sequenced by pyrosequencing. The preparation of the Paired End (PE) library followed the manufacture’s procedure and resulted in a collection of short DNA tags (approximately 3 kb and 8 kb apart in the original sample) ready to be used to the emulsion PCR and sequencing by 454 platform. The shotgun library yielded approximately 400,000 reads with an average length of 430 bp for a total of more than 170 Mbp, corresponding to an average coverage of higher than 20x. The 3- and 8-kb PE libraries produced over 130,000 and 300,000 reads, respectively (S6 Table).

From the shotgun and 3-kb PE reads, 161 large contigs were assembled into 20 scaffolds using the 454 Newbler Assembler (version 2.3, Roche). Including the reads derived from the 8-kb PE library, contigs were assembled into 10 scaffolds. The total size of the 10 scaffolds was approximately 8,69 Mbp (Table 1 and S6 Table). The genomic data are available at NCBI in Sequence reads Archive (SRA) under the accession number: Bioproject-SRP041970; Biosample-SRS606573; Experiment-SRX542125.

### Bioinformatic analyses

All annotated proteins of *P*. *rosea*, *S*. *roseum*, *Microbispora* and *S*. *coelicolor* were subject to all-vs-all BLASTP followed by Markov clustering approach to identify orthologous genes. OrthoMCL package that provides all the tools was used with the following parameters: percentMatchCutoff = 50 and evalueExponentCutoff = -5 [25, 26]. Designing of Area-Proportional 3-Venn Diagram for STPG analysis was made using EulerApe drawing tool [27]. Functional annotation of the *P*. *rosea* proteome was performed using WebMGA tool which applies the RPSBLAST algorithm (evalue = 10–5) with the COG database for prokaryotic proteins [28].

### RNA sequencing and data analysis

RNA was extracted, using GenElute total RNA Purification Kit (SIGMA), from a 1-ml mycelium pellet obtained from two separate sets of *P*.*rosea* cultures grown for 24, 30, 48, 54 and 72 h. RNA samples were extracted at the different time points from two parallel cultures and processed for sequencing to have two replicates for each bacterial cultures. After DNAse treatment, ribosomal RNAs were removed using the MICROBExpress kit (Ambion, Austin, TX). The resulting enriched mRNA (500 ng) was used for the preparation of cDNA libraries using the Ovation Prokaryotic RNA-seq System, which uses random primers designed to avoid rRNA amplification. Then, 200 ng of double stranded cDNA were used to prepare a library with NuGEN’s Encore NGS Library System, according to the manufacturer’s protocol. Due to the small size of cDNA obtained, no fragmentation treatment was performed prior to library preparation [29, 30]. Each sample was prepared as a replicate and sequenced using an Illumina Genome Analyzer IIx platform to generate paired-end 86bp reads.

Transcriptome reads were extracted using GERALD software and mapped on the *P*.*rosea* scaffolds with CLC Genomics Workbench 6.0, applying default parameters. The differential expression analysis was performed on genes covered by more than 10 reads in all the conditions analyzed, with over 90% of genes above Detection Threshold in all the time points analyzed (S3 Table). The sequencing reads produced were of high quality as evidenced by the high number of genes covered by more than 10 reads, as a matter of fact more than 90% of the genes were above this Detection Threshold in all the time points analyzed (S3 Table) The 72 h time point had quite low coverage because it was the condition in which the ribosomal RNA removal was less efficient so more reads were excluded from the analyzing because they mapped on ribosomal RNAs. Read count for gene relative abundance, differential expression analysis and statistical analysis were performed as previously described [30]. Differential expression was evaluated by using EDGER [31] performing a class comparison of sample pairs. The transcriptomic data are available at NCBI in Sequence reads Archive (SRA) under the accession number: Bioproject-SRP041970; Biosample-SRS606573; Experiment-SRX542125.

### qRT-PCR

Total RNA (1 μg), extracted as above, was reverse transcribed using the High Capacity cDNA Reverse Transcription kit (Applied Biosystems). The resulting cDNA was diluted to 5 ng/μl and 2 μl were used in each qPCR using the SYBR Green PCR Master Mix protocol and reagents (Applied Biosystems). qRT-PCR was performed by using a ABIPRISM7900 Instrument (Applied Biosystems) using the primers listed in S7 Table. All reactions were performed in three replicates from two independent cultures. As a control, we used Pros_5693, encoding sigma-70.

### Proteome analyses

Using an experimental procedure previously described [32], total proteins were extracted from frozen biomass samples collected from four parallel cultivations. Protein samples were labelled for 2D-DIGE analysis using the CyDyeTM DIGE minimal labelling kit (GE Healthcare, Sweden), according to the manufacturer's instructions as previously described [33]. Cy2 dye was used to label a pooled internal standard, consisting of equal amounts of protein extractions from the twelve different test samples, which was then used for spot co-detection and normalization of protein spot abundances. IEF was performed as previously described [33] using 3–10 non-linear pH range 18-cm IPG strips (GE Healthcare) in an Ettan IPGphor III apparatus (GE Healthcare). After IEF, IPG strips were treated as previously described [33]. The 2D-gels were scanned with a DIGE imager (GE Healthcare) according to the manufacturer’s instructions. Differential gel analysis was performed automatically using the Image Master 2D Platinum 7.0 DIGE software (GE Healthcare), according to the manufacturer’s instructions. Protein spots showing more than 1.5 fold change in normalized mean spot volume, with a statistically significant ANOVA value (P≤ 0.05), were considered differentially represented, excised from the 2D-gels and processed for protein identification as previously described [34]. Peptide mixtures were desalted and directly analyzed by nanoLC-ESI-LIT-MS/MS using a LTQ XL mass spectrometer (ThermoFisher, San Jose, CA) equipped with a Proxeon nanospray source connected to an Easy-nanoLC (Proxeon, Odense, Denmark) as reported [32]. Raw data files from the nanoLC-ESI-LIT-MS/MS experiments were searched using the MASCOT search engine (version 2.3, Matrix Science, UK) against a NCBI nonredundant database containing also the deduced *P*. *rosea* proteins. Database searching was performed using Cys carbamidomethylation and Met oxidation as fixed and variable modifications, respectively, a mass tolerance of 2.0 Da for parent ions and 0.8 Da for fragment ions, trypsin as proteolytic enzyme and a missed cleavage maximum value of 2. Candidates with at least 2 assigned peptides, with ion score >25 and an individual confidence level of 95%, were considered as properly identified.

## Supporting Information

S1 Fig Comparison of the *P*. *rosea* and *S*. *coelicolor* chromosomes. Dots represent reciprocal best hits obtained by pairwise BlastN searches.
(TIFF)

S2 Fig The *pbt* cluster and flanking genes. (TIFF)

S3 Fig *P*. *rosea* protein 2D-maps. Representative protein 2D-map of sample collected at 24 with labels referring to MS-identified protein spots (S2 Table) (A). Theoretical protein 2D-distribution calculated using the whole protein sequence data set from *P*. *rosea* genome annotation by using JVirGel on line tool [35] (B). Two representative 2D-DIGE gels obtained for differential proteomics showing co-migration of 24-h, in green, and 48-h, in red, proteins (C) and of 24h, in green, and 72h, in red, proteins (D). Both experimental and virtual maps reveal that most *P*. *rosea* proteins have a Mw ranging from 60 to 10 kDa and localize into ranges of pI 4–6.5 and 8.5–10. Only spots showing at least 1.5-fold change in normalized mean abundances with a P≤ 0.05 (ANOVA test) were considered for MS identification.
(TIFF)

S4 Fig Relative distribution into COG classes of MS-identified protein species (A panel) and protein specie abundance profile (B panel). COG classes are referred as in S2 Table.
(TIFF)

S5 Fig HeatMap representation of the 202 genes differentially expressed at all time points. Genes were clustered as defined in Fig 6. Note that the majority of DEGs decrease their expression over time.
(TIFF)

S6 Fig HeatMap representation of genes from the secondary metabolites clusters. The 464 genes covered by at least 10 reads in all conditions were analyzed by applying a hierarchical clustering, as in Fig 6. Accordingly, four major expression clusters can be identified.
(TIFF)

S7 Fig Correlation between RNASeq and RT-PCR data. Histogram representation of relative foldchanges of selected *pbt* genes. Grey and purple bars correspond to FoldChanges by RNASeq and RT-PCR, respectively.
(TIFF)

S1 Table COG distribution in *P*. *rosea*, PLBR, ACTB and STPG. COG functional categories are indicated with the number and percentage of genes in each COG category for *P*. *rosea*, PLBR, ACTB and STPG.
(DOCX)

S2 Table Description, functional classification, abundance profiles and mass spectrometry identification parameters of *P*. *rosea* proteins. (XLS)

S3 Table RNAseq statistics: total reads, mapped reads, CDS above threshold total DEGs and DEGs above filtering parameters. (XLS)

S4 Table List of all the Differentially Expressed Genes (DEGs) (p-val≤0.01 and log_2_ fold change ≥1 or log_2_ fold change ≤−1) at 30, 48, 54 and 72 h in comparison with their levels at 24 h. (XLS)

S5 Table List of all the 202 Differentially Expressed Genes (DEGs) (p-val≤0.01 and log_2_ fold change ≥1 or log_2_ fold change ≤−1) in common _and expression values. (XLS)

S6 Table Sequencing and assembly results. (DOCX)

S7 Table Oligo used for qRT-PCR. (DOCX)
