# Supplementary figures and images for: A Genomic, Transcriptomic and Proteomic Look at the GE2270 Producer Planobispora rosea, an Uncommon Actinomycete
Source: PLoS One. 2015 Jul 24;10(7):e0133705. doi: 10.1371/journal.pone.0133705 (PMC4514598; doi:10.1371/journal.pone.0133705)

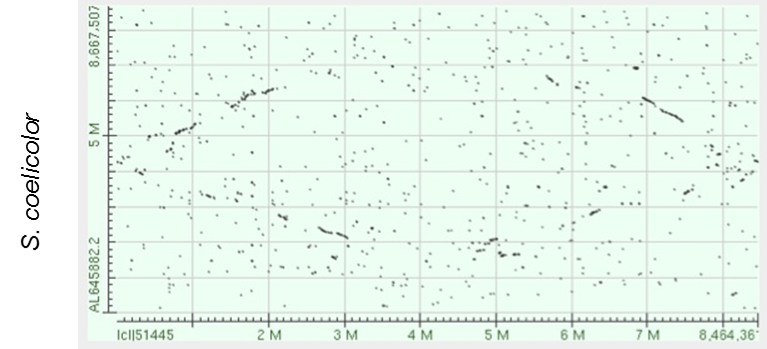

Supplement: S1 Fig — Dots represent reciprocal best hits obtained by pairwise BlastN searches. (TIFF) [file pone.0133705.s001.tiff]

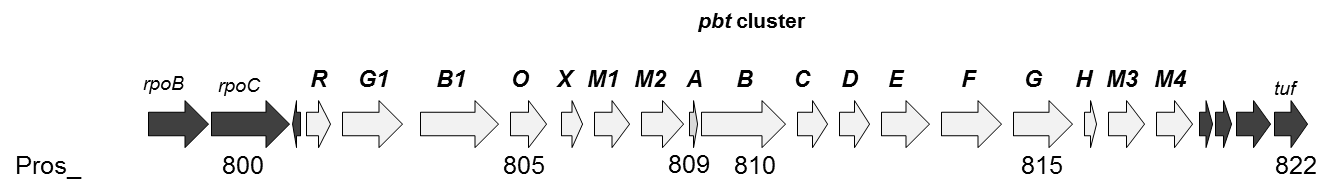

Supplement: S2 Fig — (TIFF) [file pone.0133705.s002.tiff]

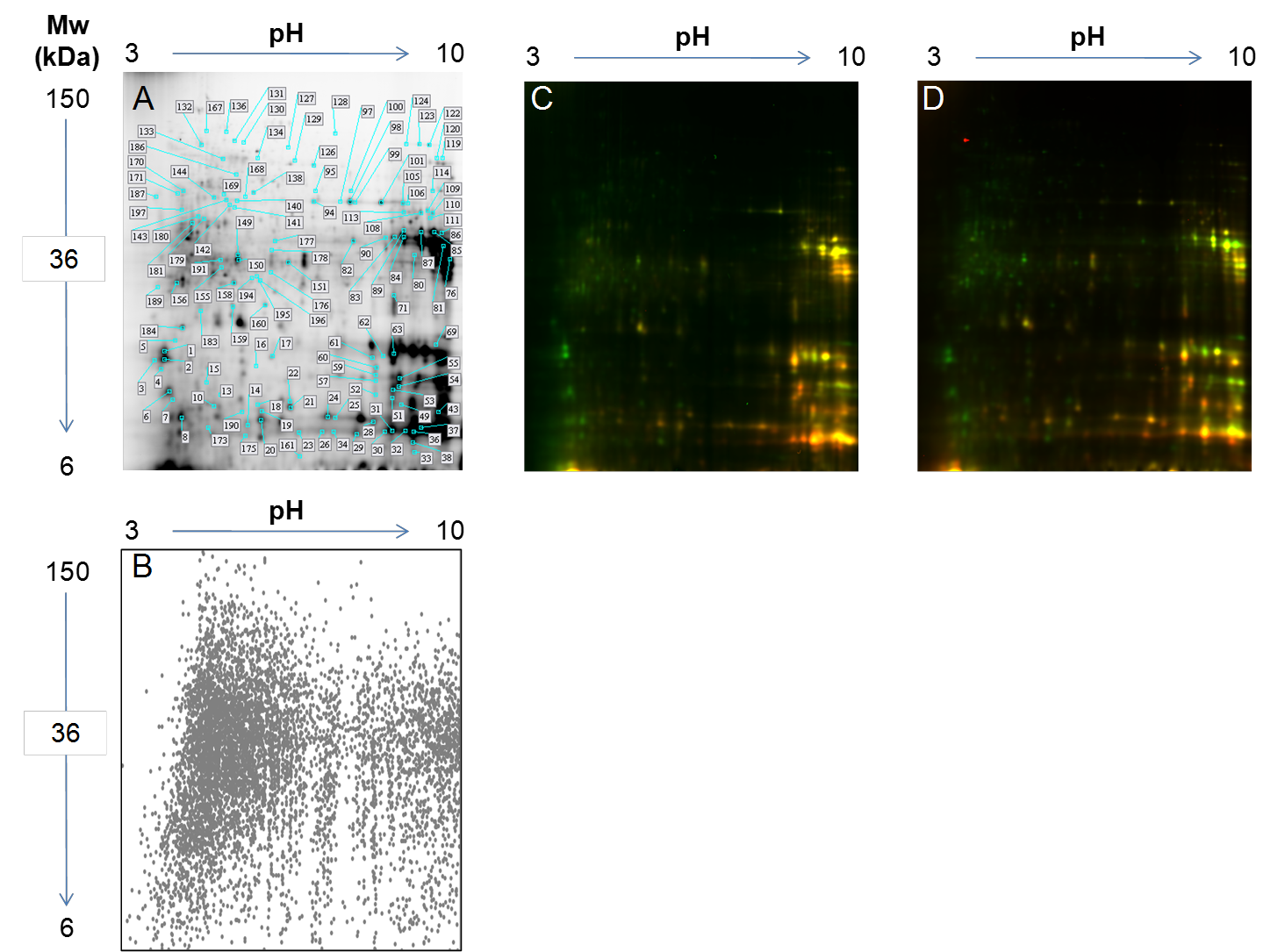

Supplement: S3 Fig — Representative protein 2D-map of sample collected at 24 with labels referring to MS-identified protein spots (S2 Table) (A). Theoretical protein 2D-distribution calculated using the whole protein sequence data set from P. rosea genome annotation by using JVirGel on line tool [35] (B). Two representative 2D-DIGE gels obtained for differential proteomics showing co-migration of 24-h, in green, and 48-h, in red, proteins (C) and of 24h, in green, and 72h, in red, proteins (D). Both experimental and virtual maps reveal that most P. rosea proteins have a Mw ranging from 60 to 10 kDa and localize into ranges of pI 4–6.5 and 8.5–10. Only spots showing at least 1.5-fold change in normalized mean abundances with a P≤ 0.05 (ANOVA test) were considered for MS identification. (TIFF) [file pone.0133705.s003.tiff]

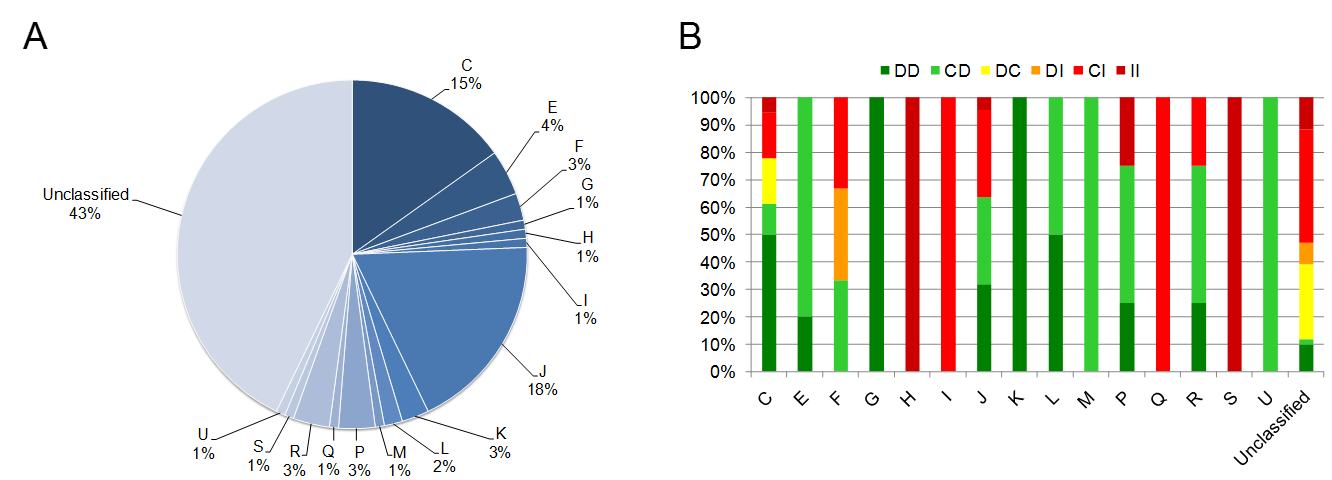

Supplement: S4 Fig — COG classes are referred as in S2 Table. (TIFF) [file pone.0133705.s004.tiff]

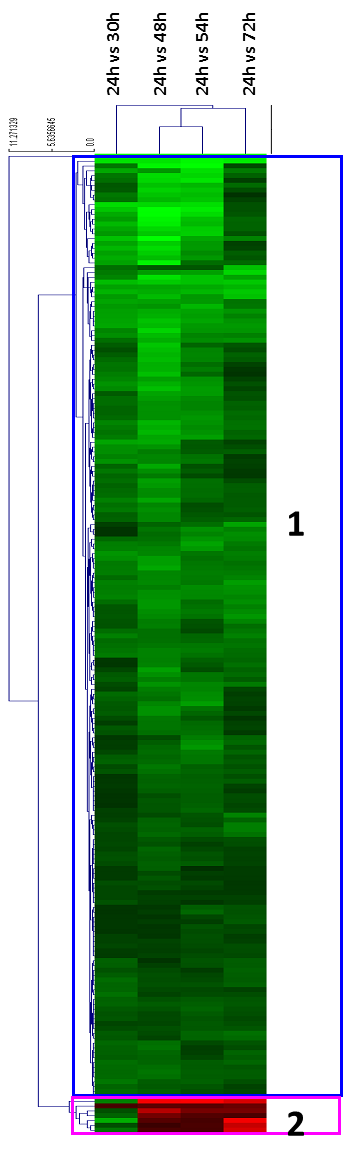

Supplement: S5 Fig — Genes were clustered as defined in Fig 6. Note that the majority of DEGs decrease their expression over time. (TIFF) [file pone.0133705.s005.tiff]

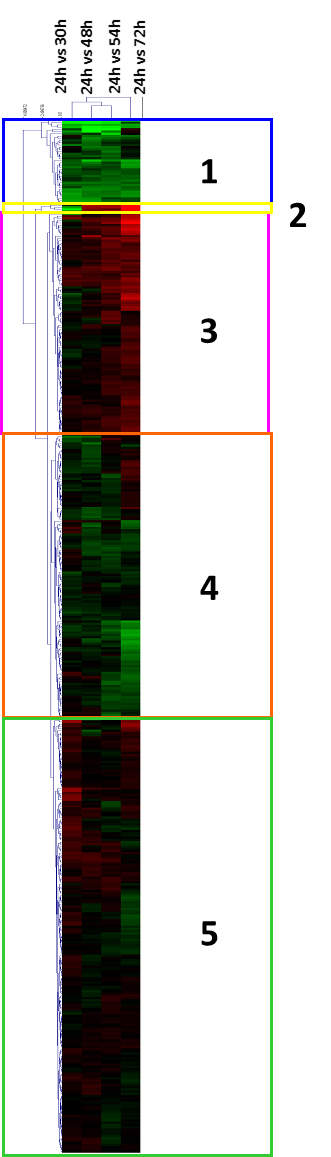

Supplement: S6 Fig — The 464 genes covered by at least 10 reads in all conditions were analyzed by applying a hierarchical clustering, as in Fig 6. Accordingly, four major expression clusters can be identified. (TIFF) [file pone.0133705.s006.tiff]

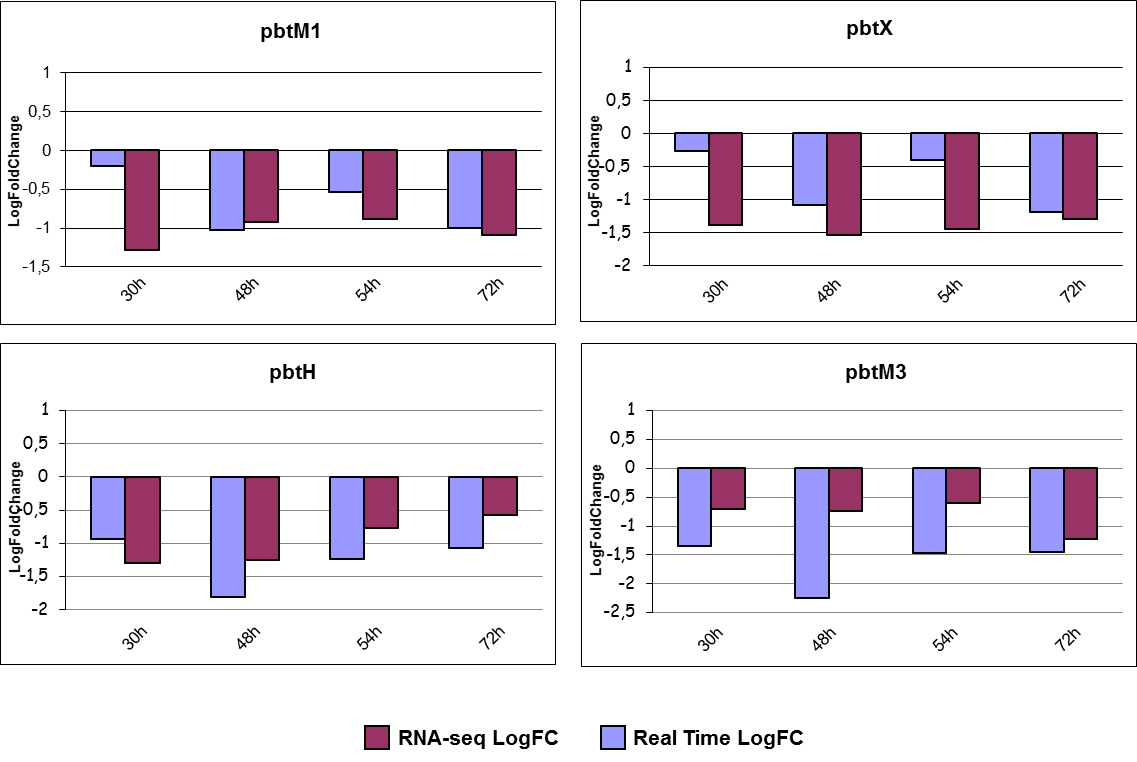

Supplement: S7 Fig — Histogram representation of relative foldchanges of selected pbt genes. Grey and purple bars correspond to FoldChanges by RNASeq and RT-PCR, respectively. (TIFF) [file pone.0133705.s007.tiff]
