# Supplementary material for: A Genomic, Transcriptomic and Proteomic Look at the GE2270 Producer Planobispora rosea, an Uncommon Actinomycete
Source: PLoS One. 2015 Jul 24;10(7):e0133705. doi: 10.1371/journal.pone.0133705 (PMC4514598; doi:10.1371/journal.pone.0133705)
Supplement: S1 Table — COG functional categories are indicated with the number and percentage of genes in each COG category for P. rosea, PLBR, ACTB and STPG. (DOCX) [file pone.0133705.s008.docx]

**Table S1** COG distribution of P. rosea CDSs.^a^

| **Clusters of Orthologous Groups** | | **total** | | **PLBR** | | **ACTB** | | **STPG** | |
| --- | --- | --- | --- | --- | --- | --- | --- | --- | --- |
| **Class** | **Description** | **N** | **%** | **N** | **%** | **N** | **%** | **N** | **%** |
| A | RNA processing and modification | 3 | 0 | 2 | 0 | 1 | 0 | 1 | 0 |
| B | Chromatin structure and dynamics | 1 | 0 | 0 | 0 | 1 | 0 | 1 | 0 |
| C | Energy production and conversion | 279 | 3 | 33 | 1 | 151 | 7 | 189 | 5 |
| D | Cell cycle control, cell division, chromosome partitioning | 43 | 1 | 11 | 0 | 19 | 1 | 23 | 1 |
| E | Amino acid transport and metabolism | 430 | 5 | 54 | 2 | 203 | 9 | 275 | 8 |
| F | Nucleotide transport and metabolism | 101 | 1 | 6 | 0 | 69 | 3 | 78 | 2 |
| G | Carbohydrate transport and metabolism | 384 | 5 | 49 | 2 | 168 | 7 | 241 | 7 |
| H | Coenzyme transport and metabolism | 197 | 2 | 21 | 1 | 100 | 4 | 127 | 4 |
| I | Lipid transport and metabolism | 254 | 3 | 31 | 1 | 93 | 4 | 151 | 4 |
| J | Translation, ribosomal structure and biogenesis | 192 | 2 | 10 | 0 | 140 | 6 | 158 | 4 |
| K | Transcription | 559 | 7 | 68 | 3 | 187 | 8 | 302 | 8 |
| L | Replication, recombination and repair | 166 | 2 | 31 | 1 | 84 | 4 | 97 | 3 |
| M | Cell wall/membrane/envelope biogenesis | 237 | 3 | 37 | 2 | 87 | 4 | 144 | 4 |
| N | Cell motility | 111 | 1 | 104 | 4 | 0 | 0 | 2 | 0 |
| O | Posttranslational modification, protein turnover, chaperones | 139 | 2 | 12 | 0 | 72 | 3 | 93 | 3 |
| P | Inorganic ion transport and metabolism | 188 | 2 | 32 | 1 | 62 | 3 | 85 | 2 |
| Q | Secondary metabolites biosynthesis, transport and catabolism | 155 | 2 | 23 | 1 | 30 | 1 | 54 | 1 |
| R | General function prediction only | 676 | 8 | 112 | 5 | 225 | 10 | 362 | 10 |
| S | Function unknown | 364 | 5 | 56 | 2 | 117 | 5 | 205 | 6 |
| T | Signal transduction mechanisms | 452 | 6 | 162 | 7 | 102 | 4 | 174 | 5 |
| U | Intracellular trafficking, secretion, and vesicular transport | 42 | 1 | 13 | 1 | 13 | 1 | 20 | 1 |
| V | Defense mechanisms | 163 | 2 | 29 | 1 | 41 | 2 | 66 | 2 |
| Z | Cytoskeleton | 0 | 0 | 0 | 0 | 0 | 0 | 0 | 0 |
|  | Unclassified | 2935 | 36 | 1515 | 63 | 302 | 13 | 768 | 21 |
|  | Total CDSs | 8071 | 100 | 2411 | 100 | 2267 | 100 | 3616 | 100 |

.

**^a^** COG established as described under Material and Methods. *N* and *%* refer to total number and relative percentage, respectively, in each category. *PLBR, ACTB*  and *STPG* are defined in text.
