# Supplementary material for: A Genomic, Transcriptomic and Proteomic Look at the GE2270 Producer Planobispora rosea, an Uncommon Actinomycete
Source: PLoS One. 2015 Jul 24;10(7):e0133705. doi: 10.1371/journal.pone.0133705 (PMC4514598; doi:10.1371/journal.pone.0133705)
Supplement: S6 Table — (DOCX) [file pone.0133705.s013.docx]

**Table S6** Sequencing and assembly results

|  | Shotgun Library | 3k Pe Library | 8k Pe Library |
| --- | --- | --- | --- |
| N reads | 400,000 | 130,000 | 300,000 |
| Average read length (bp) | 430 | 230 | 330 |
| N contigs | 198 | 161 | 117 |
