# Supplementary material for: A Genomic, Transcriptomic and Proteomic Look at the GE2270 Producer Planobispora rosea, an Uncommon Actinomycete
Source: PLoS One. 2015 Jul 24;10(7):e0133705. doi: 10.1371/journal.pone.0133705 (PMC4514598; doi:10.1371/journal.pone.0133705)
Supplement: S7 Table — (DOCX) [file pone.0133705.s014.docx]

**Table S7** Oligo used for qRT-PCR

| **CDS** | **Sequence (5’-3’)** |
| --- | --- |
| Pros_0799 | GATGTTCGTCACGGCGGA; CTTGATCTCGCCGGTGGT |
| Pros_0800 | TCCAACTTCGACCTCGACG; TGCGGATCGTCTCGCGCA |
| Pros_0802 | AGCGCTGCAGAAAAACCAAC; GTGATGCGCAGATCGATGTC |
| Pros_0803 | GAGCATGGCCGGTGCGAT; CACCAGGTCCAGTGCCG |
| Pros_0806 | ATCAGGCATTCGACCGTCC; GACAGTTTCCCGACGAAAG |
| Pros_0807 | TGTGGCTGCCCAACCTCAT; TGGCACCACAGCCACTGG |
| Pros_0809 | GAAGTCGAGTCACTCACCG; CGCGGACGGGCTGCAGG |
| Pros_0810 | AAAGGTGCTCAGACTCGTCAAATA; CCATGAAGGAGCCGAACG |
| Pros_0811 | ACCACATGAACGCGTACTTC; TGGGTCATGGTCTCCGGT |
| Pros_0816 | GGACGATTACTCCAGGCTG; TCGGAGAACCACGCCTTG |
| Pros_0817 | GAGCCGTTGCTGCTCTACGT; GTGGCATTTGGAGTTGCAGA |
| Pros_0822 | CCGATCGAGGACGTCTTCTC; CTCACGGATCGCGAACTTG |
| Pros_5693 | ACACCAAGGGTTACAAGTTCTCCA; CCTGCCGGATCCACCAC |
